# Management-supportive measures for managers of healthcare organizations during the COVID-19 epidemic

**DOI:** 10.1017/ice.2020.108

**Published:** 2020-04-06

**Authors:** Reza Dehnavieh, Khalil Kalavani

**Affiliations:** 1 Faculty of Management, Kerman University of Medical Sciences Kerman, Kerman, Iran; 2 Health Services Management, Faculty of Management, Kerman University of Medical Sciences Kerman, Kerman, Iran

*To the Editor*—Contagious or infectious diseases are a major cause of death.^1^ Epidemics are a serious threat to public health and a global challenge,^2^ and the management of these epidemics is very difficult. In these conditions, economic, social, and health factors of the country are of utmost concern; therefore, healthcare managers must properly manage and support healthcare centers^3^ and use supportive measures for the organization and staff to provide the best healthcare services possible. Presenting a scientific framework for managing health centers can be very helpful. The following management-supportive practices are the most important at healthcare centers during outbreaks:

1. Engage leadership: Leadership affects the performance of physicians and nurses. Maintain effective communication with employees, pay attention to them, and listen to them effectively.
2. Choose wise motivations: Talk about the importance of staff work, appreciate their work, and provide encouragement.
3. Note work–life balance: Define a proper and balanced workload for employees. Say that optimal performance depends on enough rest and emphasize the need to re-energize.
4. Encourage peer support: Protect your staff from external pressures and from illogical or uncertain demands from patients and individuals, and promote support among colleagues.
5. Provide resources to protect employees and their mental health: Minimize risky conditions in the organization and minimize workplace stress to ensure that staff are not exposed to additional stressors.
6. Build a good community: Build the right teamwork and improve working relationships.
7. Increase employee control over their work: Clarify your expectations of employees and create an environment for team members to perform important tasks without interruption.
8. Review your achievements regularly: Talk to staff about progress and successes.
9. Cancel unnecessary meetings: Try to avoid unnecessary gatherings at work. Use video conferencing if a meeting needs to be held.
